# Improving Advanced Communication Skills Towards the Family System: A Scoping Review of Family Meeting Training in Oncology and Other Healthcare Settings

**DOI:** 10.3390/cancers17193115

**Published:** 2025-09-24

**Authors:** Sara Alquati, Loredana Buonaccorso, Nuria Maria Asensio Sierra, Francesca Sassi, Francesco Venturelli, Maria Chiara Bassi, Stefano David Scialpi, Silvia Tanzi

**Affiliations:** 1 Palliative Care Unit, Azienda USL-IRCCS di Reggio Emilia, 42123 Reggio Emilia, Italy; sara.alquati@ausl.re.it (S.A.); francesca.sassi2@ausl.re.it (F.S.); silvia.tanzi@ausl.re.it (S.T.); 2 Psycho-Oncology Unit, Azienda USL-IRCCS di Reggio Emilia, 42121 Reggio Emilia, Italy; 3 Medical Oncology Unit, University Hospital of Parma, 43126 Parma, Italy; asensio@ao.pr.it; 4 Epidemiology Unit, Azienda USL-IRCCS di Reggio Emilia, 42122 Reggio Emilia, Italy; francesco.venturelli@ausl.re.it; 5 Medical Library, Azienda USL-IRCCS di Reggio Emilia, 42123 Reggio Emilia, Italy; mariachiara.bassi@ausl.re.it; 6 Primary Health Care and Community, University of Modena and Reggio Emilia, 41121 Modena, Italy; stefanodavid.scialpi@ausl.re.it

**Keywords:** family meeting, palliative care, oncology, education, training, communications, systematic review, scoping review

## Abstract

**Background/Objectives:** Family meetings (FMs) are clinical encounters in a structured space between the patient, family members, and care teams. Healthcare professionals (HPs) often lack formal training in conducting FMs. The scoping review aims to provide an overview of the available research evidence on FMs’ education for HPs. **Methods**: We searched MEDLINE, Embase, CINAHL, PsycINFO, and Scopus. The PCC (Population-Concept-Context) framework was used to define inclusion criteria: educational intervention on FMs aimed at HPs in all settings of care and students of medicine and nursing sciences treating adult patients with oncological and non-oncological diseases. **Results:** The search retrieved 1017 articles, of which 26 were eligible. The training had as its primary aims the development of communication skills and curriculum development/evaluation. For the most part, palliative care physicians served as trainers, while medical students and residents represented a major part of trainees, underscoring a focus on early-career learners. FM training is mainly provided in the American countries and intensive care settings. Role-play or simulation was the most common teaching method. Pre- and post-interventional designs were the most common, with few studies incorporating longitudinal follow-up to assess skill retention. Quantitative and qualitative methodologies were used to evaluate interventions. **Conclusions:** The training topics are related to advanced communication, but there is a lack of an interprofessional perspective and long-term assessment of the skills learned. It is necessary to consider different family types as subjects of communication.

## Simple Summary

This review aimed to provide an overview of the available research evidence on family meetings education for healthcare professionals. We discussed some specific findings that emerged from our data, which we believe can guide clinicians in designing effective interventions. Data revealed that communication skills training dominated the literature. The topic of communication skills is directly related to the theoretical frameworks of the training, which emphasize the importance of empathy and active listening in communicating and supporting the family system. The training topics are related to advanced communication, but there is a lack of an interprofessional perspective and long-term assessment of the skills learned. According to clinical practice guidelines, family meetings should be conducted by a multiprofessional team, including a physician and a nurse or another key figure involved in patient/family care. It is therefore essential for healthcare professionals to assess the family system they will support. Evaluating these topics helps clinicians offer personalized care, intercepting families with dysfunctional communication styles, so they can work preventively to activate the specialists.

## 1. Background

Effective communication between patients, families, and healthcare professionals (HPs) is essential for high-quality care [1]. The literature indicates that honest and open communication with cancer patients can improve adherence to treatment programs [2,3] and benefit physicians [4]. Family meetings (FMs) are clinical encounters in a structured space involving the patient, their family, and caregivers conducted for multiple purposes, including sharing information and concerns, clarifications regarding goals of care, discussions on diagnosis, treatment, prognosis, and the development of a plan for the care and assistance of the patient and their family members [5,6,7].

FMs have been shown to improve concordance of care with expressed wishes and to reduce post-traumatic stress disorder, anxiety, and depression among bereaved family members [8,9]. They are also associated with reduced length of inpatient stay and higher quality ratings of the dying experience [10]. Although FMs are often used in the patient care pathway, data show how infrequently family members’ concerns are addressed as a fundamental element of family care, which indirectly affects patient care [11,12,13].

The literature shows that most HPs do not receive specific training and consequently do not feel adequately prepared to participate, which can accentuate the challenges of conducting them effectively [11,12]. It is widely documented that developing and implementing communication training courses is necessary for hospital HPs, since difficult conversations frequently occur in hospital settings, and all HPs should receive specific training [2,6,14,15,16].

This scoping review aims to provide an overview of the available research evidence on FMs’ education for HPs.

## 2. Methods

The review question was “How are training programs on family meetings for healthcare professionals designed, organized and evaluated?”. Following the Arksey and O’Malley framework [17], our scoping review process comprised five key phases: research question formulation, study identification and selection, data charting, data collection and synthesis, and results reporting. This review was reported following the Preferred Reporting Items for Systematic Reviews and Meta-Analyses-extension for Scoping Review (PRISMA-ScR) guidelines [18]. The PCC (Population-Concept-Context) framework was used to structure the search strategy and to define the inclusion criteria [19].

Review protocol has been registered in the OSF registry (https://doi.org/10.17605/OSF.IO/2Y836).

### 2.1. Search Strategy

We conducted an electronic search of the literature from inception up to 28 February 2025 on the following databases: MEDLINE (through PubMed), Embase, CINAHL, PsycINFO, and Scopus. We included articles in English, Italian, and Spanish. The search strategy employed the following terms: (‘family meeting’[Title/Abstract] OR ‘family conference’[Title/Abstract]) AND (education* OR learning OR training OR course* OR workshop* OR teaching OR seminar* OR class* OR instruction). No additional searching was conducted, but we screened the reference list of included articles to identify additional relevant publications.

### 2.2. Inclusion Criteria

We included studies that described an educational intervention on FMs (Concept) aimed at HPs in all settings of care and students of medicine and nursing sciences (Population) treating adult patients (>18 years old) with oncological and non-oncological diseases (Context).

We adopted a comprehensive approach to identify eligible educational intervention, starting from Moneymaker’s definition of FM [5]: “The Family Meeting are meeting between the patient, their family and healthcare professionals are undertaken for multiple purposes including the sharing of information and concerns, clarifying the goals of care, discussing diagnosis, treatment, prognosis and developing a plan of care for the patient and family carers”. The eligible interventions included FMs and family conference training. We included studies utilizing diverse research methodologies, encompassing quantitative, qualitative, and mixed methods programs. Outcomes were described when available in the included studies to provide information about how training was structured and evaluated.

### 2.3. Exclusion Criteria

We excluded studies if they (1) described an educational intervention on advanced communication without a specific focus on FMs; (2) included only pediatric settings or pediatric HPs. We also excluded conference abstracts, case reports, systematic reviews, expert opinions, guidelines, ongoing trials, protocol articles, and book chapters.

### 2.4. Screening and Selection of Studies

**Figure 1:**
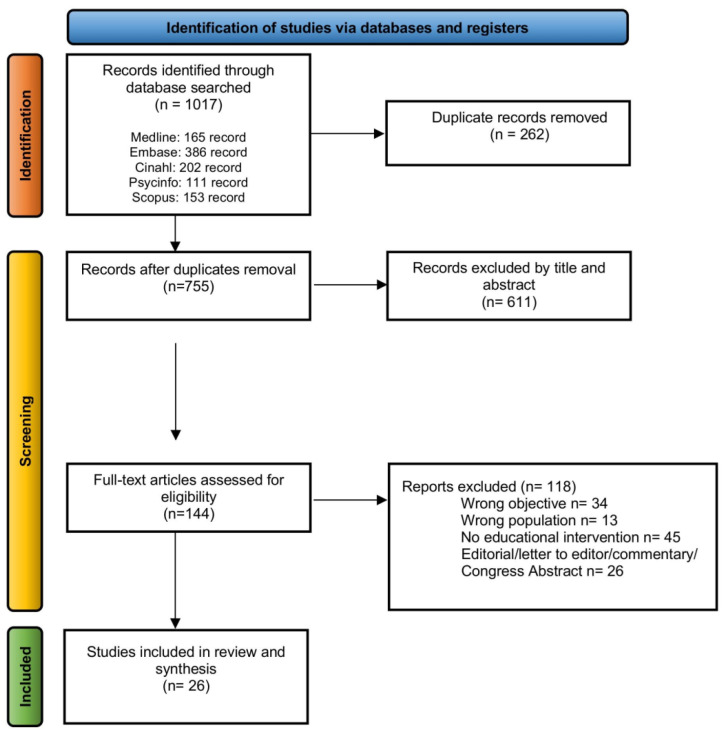
Flow chart scoping review process.

Four authors (S.A., L.B., A.S.N.M., F.S.) independently screened titles and abstracts on inclusion and exclusion criteria. After multiple screening rounds, a 100% agreement was reached on articles judged to be eligible for full-text examination. All potentially eligible articles were independently examined in full text by four authors working in two evaluation groups. Final eligibility decisions required 100% consensus, with a supervising author (ST) consulted to resolve any discrepancies. The results of the study selection process are summarized in the flow chart (Figure 1).

### 2.5. Data Extraction

Two reviewers (SA and LB) performed data extraction from included full-text articles using a data extraction tool developed by the working group. The data extraction form included the first author’s name, year of publication, country, objective, study design, trainers, trainees, setting and duration of training, delivery of intervention, type/program of training, quantitative evaluation tools, qualitative evaluation, quantitative and qualitative outcomes, and refeence to the definition of FM (Table 1).

### 2.6. Quality Assessment of Included Studies

Given the descriptive aims of the scoping review, according to Johann Briggs institute guidance for scoping review, it was deemed that the assessment of the quality of included studies was not needed [19].

### 2.7. Synthesis of the Results

**Figure 2:**
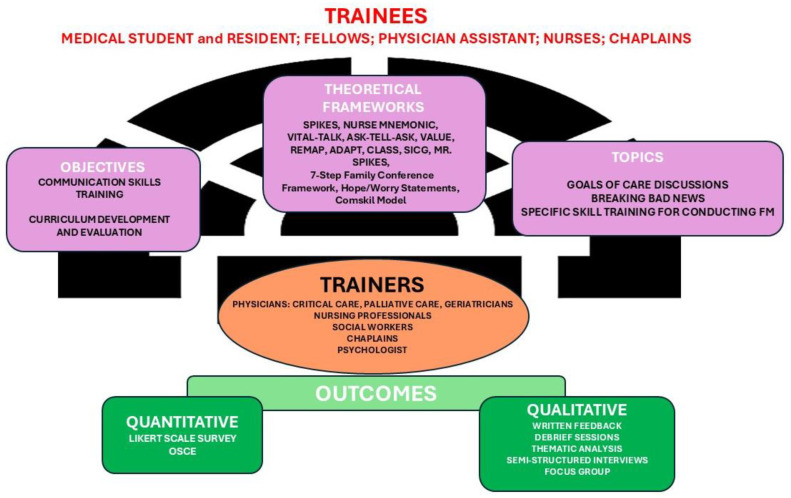
The highlighting characteristics of the interventions.

**Figure 3:**
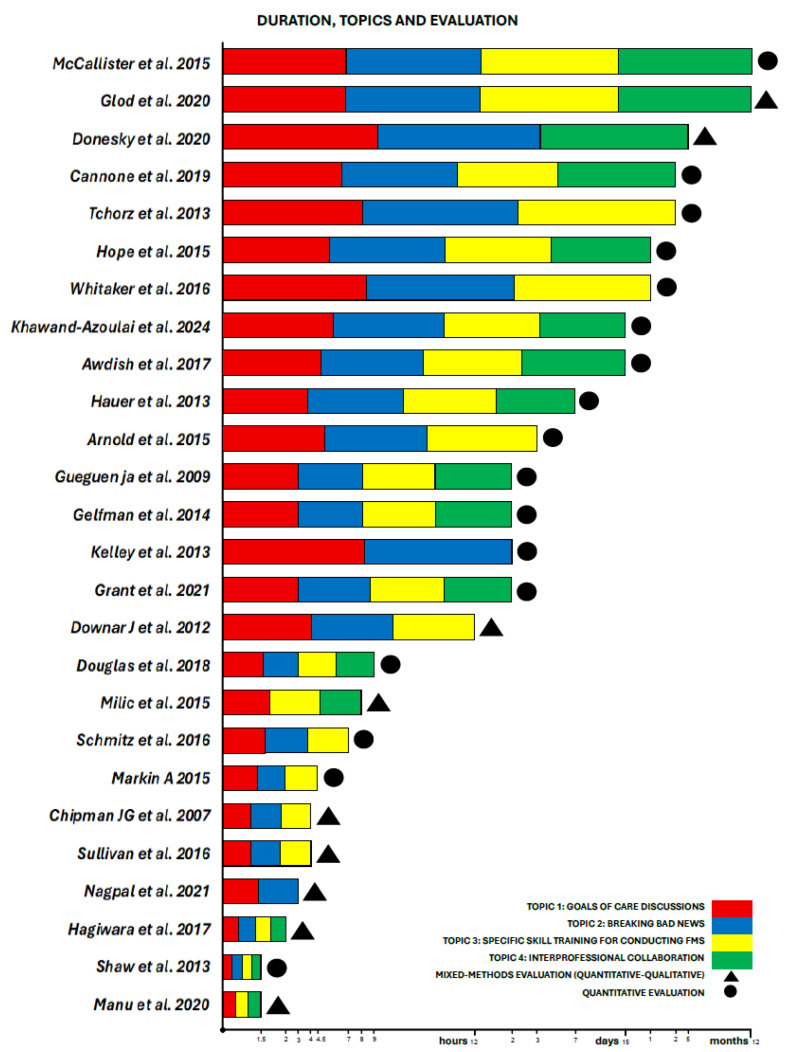
Duration, topics and evaluation.

The results from the included studies were reported according to the following topics: Study Objective, Study Design, Trainers, Trainees, Setting of Training, Intervention (Duration, Teaching Methods, Theoretical Framework), Topics, and Evaluation of Training (Quantitative Evaluation Tools and Outcomes, Qualitative Evaluation and Outcomes). In Figure 2, we summarized the articles included in the scoping review, highlighting the principal characteristics of the intervention: -objectives, theoretical frameworks and the topics; -trainers and trainees; -outcomes. In Figure 3, we summarized the highlighting characteristics of the analyzed studies.

## 3. Results

The search yielded 1017 records, which were reduced to 755 after duplicate removal. Initial title/abstract screening excluded 611 more articles. Of the 144 full-text articles retained for further screening, 121 were discarded primarily because the sample did not involve HPs or students, or lacked focus on FMs training. Other papers were excluded because of editorials, letters to the editor, commentaries, and congress abstracts (Figure 1). Twenty-six articles were ultimately included in the scoping review (Table 1).

Geographically, studies focused on North America, with only one Canadian study [43] and no European studies. The training had as its primary aims the communication skills [20,21,22,23,26,27,28,29,30,31,32,33,34,35,36,37,38,39,41,42,43,44,45] and curriculum development/evaluation [43,44,45]. For the most part, palliative care physicians served as trainers [20,22,24,25,26,27,29,33,34,36,37,38,39,40]. Studies predominantly targeted physicians, particularly medical students. Only four studies involved nurses. The role-play and simulation were the most common delivery methods. Quantitative methods predominated, with a smaller proportion [22,33,35,38,43] incorporating longitudinal follow-up to assess skill retention.

### 3.1. Study Objective

The systematic analysis revealed two primary aims: (i) communication skills training and (ii) curriculum development and evaluation.

(i) Communication Skills Training [20,21,22,23,26,27,28,29,30,31,32,33,34,35,36,37,38,39,41,42,43,44,45]

Studies in this category aimed to enhance specific communication competencies, including delivering bad news or prognosis [33], conducting FMs [29], and discussing goals of care [22]. For example, Khawand-Azoulai et al. [20] focused on end-of-life discussions for medical students; Sullivan et al. [31] targeted Intensive Care Unit (ICU) residents’ skills in family communication; and Chipman et al. [45] developed an Objective Structured Clinical Examination (OSCE) to assess surgical residents’ ability to lead family conferences and formalized assessment tools to evaluate surgical residents’ conference leadership skills. Training emphasized collaborative skills across disciplines. For example, Grant et al. [21] trained physician assistant students and chaplains jointly to address spiritual dissonance during prognosis delivery; Milic et al. [37] empowered nurses to engage in interdisciplinary prognosis discussions.

(ii) Curriculum Development and Evaluation [43,44,45]

These studies piloted or assessed novel curricula: Kelley et al. [40] evaluated a retreat-style course for geriatrics fellows; Glod et al. [24] designed a hybrid ICU FMs curriculum integrating didactics with real-world facilitation; while Manu et al. [25] tested a seminar format for dementia-related communication.

### 3.2. Trainers

Physicians served as trainers in 24 of the 26 studies reviewed [20,21,22,24,25,26,27,28,29,30,31,32,33,34,35,36,37,39,40,41,42,43,44,45]. For the most part, they were palliative care physicians [22,25,26,27,29,30,31,33,34,39,40,43,44,45], followed by critical care specialists [24,27,28,31,32,33,34,36,37,43,45]. Nursing professionals participated explicitly in seven studies [20,24,27,28,34,37,45] of training programs. Other HPs included social workers [20,24], chaplains [21,37], and psychologists [20,36,37].

Most studies reported multiprofessional training teams [20,21,22,24,25,28,30,31,33,34,36,37,38,39,40,41,42,43,44,45], for example physician-and-nurse trainer teams [20,24,28,31,34,37,38,43,45].

### 3.3. Trainees

A total of 1914 trainees participated across 26 studies. Medical students represented the largest cohort (*n* = 851), driven by three studies: Hagiwara et al. (*n* = 674) [29], Tchorz et al. (*n* = 97) [42], and Khawand-Azoulai et al. (*n* = 80) [20]. Trainee roles exhibited significant heterogeneity across disciplines. Medical residents constituted the second-largest group [20,23,26,27,28,32,35,36,37,39,40,41,44,45], primarily from internal medicine disciplines [22,25,31,32,35,39], with additional representation from surgery [30,45] and neurology [25,27]. Fellows and physician assistant students were prominent in critical care and inter-professional contexts [21,44]. Internal medicine was the most common specialization, primarily made up of residents engaged in ICU rotations [24,31,32,35,39] or palliative care training [24,35,39]. Critical care included fellows [33,34,36,43], nurses [37,41] and residents, reflecting the ICU context of most interventions [24,28,31,32,33,34,35,36,37,41,43,45]. Other HPs included chaplains [21,23,41].

### 3.4. Setting of Training

The distribution of training settings reveals a pronounced emphasis on clinical environments: ICU [23,28,31,32,33,37,41,42,44] and non-ICU [20,22,25,30,39,44]. Simulation centers also emerged as a setting [21,22,23,26,44,45] providing controlled environments for high-stakes communication practice. Also, exclusively academic-hosted studies were reported, primarily using classrooms or lecture halls [21,22,26,29,45].

A distinct set of studies [21,24,25,26,31,32,33,35,36,37,38,39,41,42,44,45] adopted mixed settings, blending simulation with clinical or academic environments. Only one study [33] utilized a specialized non-clinical, off-site retreat setting for ICU fellows (no more specifics provided). Direct involvement of oncology staff (trainees or trainers) resulted in rare cases, with only a few exceptions where oncology intersects with palliative care or surgical training. Only two studies explicitly include oncology professionals as trainees: -Cannone et al. [26] trained oncology fellows and radiation oncology residents at the Center for Learning and Innovation in Lake Success, NY—a setting affiliated with cancer and palliative care programs; -Gueguen et al. [44] involved multi-specialty professionals, including medical, surgical, and radiation oncologists, in a communication skills workshop at Memorial Sloan-Kettering Cancer Center in New York.

### 3.5. Intervention

We analyzed the type of intervention through three principal topics: (i) duration; (ii) teaching methods; and (iii) theoretical framework.

(i) Duration

The data demonstrate a preference for longitudinal training formats [20,23,24,26,32,34,36,39,42], with some programs spanning weeks to months, exemplified by McCallister’s [36] year-long pulmonary fellowship curriculum and Tchorz’s [42] eight-week surgical clerkship incorporating structured clinical examinations. Shorter interventions were distributed between intensive single-day workshops [21,23,25,37,41,43], including Milic’s [37] 8 h nursing communication training, and focused multi-day retreats [33,38,40,43,44], such as Arnold’s [33] three-day intensive care fellowship program. Compact 1–4 h sessions accounted for the other interventions [20,22,24,27,29,31,35], typified by Nagpal’s [22] 3 h simulation for internal medicine residents and Sullivan’s [31] 4 h ICU communication workshop.

(ii) Teaching Methods

The pedagogical approach highlighted a preference for active learning methodologies. Simulation-based training dominates, employed in 24 studies through various formats including standardized patient encounters, role-playing exercises, and high-fidelity clinical scenarios [20,21,22,23,24,25,26,27,28,29,30,31,32,33,34,35,36,37,38,40,41,42,43,44,45]. This experiential approach frequently combines with other methods in blended designs, creating multimodal learning experiences that integrate didactic instruction with hands-on practice and clinical application [21,22,25,26,27,28,29,30,31,32,33,34,35,37,38,40,43,44,45].

(iii) Theoretical Framework

The most frequently cited model was SPIKES [46], a six-step protocol for delivering bad news, which appeared in 11 out of 26 articles [20,24,26,27,29,34,36,37,38,41,43]. This model emphasizes structured communication through the steps of Setting, Perception, Invitation, Knowledge, Emotions, and Strategy/Summary. The NURSE mnemonic, designed to address emotional responses with the steps Name, Understand, Respect, Support, and Explore, was the second most prevalent, appearing in eight articles [20,24,27,34,35,36,37,38]. Other notable models included VitalTalk and Ask-Tell-Ask (ATA), each cited in five articles [22,23,24,27,28,32,35,36,37]. VitalTalk focuses on structured communication for serious illness discussions, while ATA is a technique for clarifying understanding by asking, explaining, and verifying comprehension.

Less frequently cited models included VALUE [24,36,43], a framework for family-centered communication; REMAP (two articles) [20,24], a protocol for care goal discussions; and ADAPT [20], CLASS [20], Serious Illness Conversation Guide (SICG) [22], MR. SPIKES [26], 7-Step Family Conference Framework [40], Hope/Worry Statements [37], and Comskil Model [44] are present as unique models.

These models address diverse aspects of communication, such as prognosis discussions, general communicative strategies, and family conferences. In particular, our analysis revealed that the training focused on the following topics:

- Goals of Care Discussions: It was universally present across all studies, underscoring its critical role in patient-centered care. The discussions often focused on aligning treatment plans with patient preferences and values.
- Breaking Bad News: Reported in 24 out of 26 of the studies [20,21,23,24,25,26,27,28,29,30,31,32,33,34,35,36,37,38,39,40,41,42,43,44], this theme highlighted the challenges and strategies involved in delivering difficult diagnoses or prognoses. Studies such as Khawand-Azoulai et al. [20], Grant et al. [21], and Nagpal et al. [22] emphasized the importance of sensitivity and clarity in these conversations. Moreover, the prognostication was frequently tied to discussions about disease trajectories and treatment expectations. “Code Status Discussions-Do Not Resuscitate/Intubate (DNR/DNI)” was present in ten studies [25,28,30,32,34,36,37,38,42,43]; these discussions were often framed as part of broader goals of care conversations, in critical or end-of-life scenarios. Seven studies focused on the challenges and strategies for transitioning patients to hospice care, often involving sensitive discussions about prognosis and quality of life [22,29,33,35,38,42,44]. Finally, in five studies [20,33,34,40,43], discontinuing life-sustaining treatments involved ethically and emotionally complex decisions.
- Specific skill training for conducting FMs: A significant majority of studies (23/26) [20,22,23,24,25,27,28,29,30,31,32,33,34,35,36,37,38,39,40,41,42,44,45] addressing preparation for conducting FMs emphasized the value of preparation before difficult conversations (pre-meeting), including reviewing patient history and anticipating family concerns. Furthermore, conflict mediation was identified as a critical skill for resolving disagreements among families or between families and healthcare teams. All studies addressed the necessity of empathy in healthcare communication. Moreover, the importance of active listening is discussed in 22 of the studies [20,22,23,24,25,26,27,28,29,31,32,33,34,35,36,37,39,40,41,42,43,44], with a focus on its role in understanding patient concerns.

### 3.6. Evaluation of Training

We presented a summary of two principal types of evaluation and outcome: (i) quantitative evaluation tools and outcomes, and (ii) qualitative evaluations and outcomes.

(i) Quantitative evaluation tools and outcomes

Quantitative methodologies are represented in all studies. Likert scale surveys were the most common tool [20,22,24,25,28,29,30,31,32,34,35,36,38,39,41,43,45], followed by Objective Structured Clinical Examinations (OSCEs) [26,29,30,42,45]. Patient/family satisfaction surveys and feedback were used in two studies [22,28]. Three unique tools—Family Meeting Communication Assessment Tool (FaMCAT) [38], JeffSATIC [23], and Mini-CEX [22]—were each used once.

A significant subset adopted mixed-methods approaches [22,23,24,25,29,31,37,43,45], integrating quantitative metrics with qualitative analysis. For instance, Nagpal et al. [22] combined pre/post surveys with thematic analysis of trainee feedback, while Sullivan et al. [31] supplemented quantitative ratings with family member interviews to capture communication preferences. Analysis of quantitative outcomes demonstrated confidence in communication as the predominant measured theme (16/26 studies) [20,21,22,23,24,26,27,28,30,31,33,34,38,40,43,44], primarily assessed via Likert-scale surveys. Communication and behavioral skill measurements represented the second most common outcome [20,21,22,23,27,28,30,32,33,34,37,40,43,44,45], utilizing OSCEs or checklists.

Interprofessional collaboration outcomes were present in six studies [20,21,23,37,39,44].

(ii) Qualitative evaluations and outcomes

Qualitative analysis was conducted in nine studies [22,23,24,25,29,31,37,43,45]. Qualitative evaluation tools were employed to assess various aspects of the interventions, spanning from feedback on used frameworks to interprofessional dialog. The most frequently used method was written feedback (e.g., open-ended survey responses or comments) [22,23,24,29,31,43]. Debrief sessions were the second most common tool [22,29,43,45], followed by thematic analysis of qualitative [23,25,29,37]. Less frequently used methods included semi-structured interviews with participants or family members [31] and focus groups [37]. 

## 4. Discussion

Of the 1017 titles screened, we included 26 articles. We discuss some specific findings that emerged from our data, which we believe can guide HPs in designing effective interventions.

A notable observation was the predominance of the North American studies, while no studies were conducted in Europe. The identified training programs appeared to minimize cultural differences; however, training on FMs should be proposed based on cultural specificity, because the goals of care, the values and desires of patients, and the communication of bad news can take on different meanings depending on the culture of belonging. By integrating cultural competence into training curricula, HPs can enhance their capacity to deliver relational support to the family system that respects the unique cultural background of patients, promoting more patient-centered healthcare practices, as suggested by other studies [47,48,49].

The analysis revealed that communication skills training dominated the literature [20,21,22,23,24,25,26,27,28,30,31,33,34,37,38,40,43,44,45]. These studies prioritized competencies such as delivering difficult prognoses, facilitating FMs, and navigating end-of-life discussions. Data suggested that communication on bad news cannot be improvised because the FMs are also a tool for supporting patients and families, not just for giving technical information. Inadequate communication could have negative effects, resulting in psychological distress due to unmet needs, lack of decision-making, and mistrust of HPs [9]. According to the family systems approach, each family member is interdependent on the others to cope with the disease. The different coping strategies of the family system depend on the life cycle stage they are going through: young couples without children; families with small children or teenagers; families with adult children; families with elderly parents. It is therefore essential for HPs to assess the family system they will support [50]. Few of the analyzed studies reported specific training on recognizing family dynamics. Evaluating these topics helps HPs offer personalized care, intercepting families with dysfunctional communication styles so they can work preventively to activate the specialists [15,51]. For example, Kissane et al. proposed a focus on assessing aspects of family-level functioning (e.g., communication, conflict, cohesiveness) that may have been changed by cancer [9]. In general, families with a major sense of coherence, a construct referring to individual family members’ capacity to make sense of the illness and develop a coherent and meaningful narrative about its implications, report less psycho-social distress. Future training on FMs should also consider the most important information about the type of family.

According to clinical practice guidelines [52], FMs should be conducted by a multiprofessional team, including a physician and a nurse or another key figure involved in patient/family care. However, our review reveals that few educational interventions have been designed for teams (physicians and nurses) [21,23,37,39,44]. A smaller but significant subset (*n* = 5/26) focused on interprofessional training, emphasizing collaborative communication across disciplines. These studies highlighted role clarity and shared decision-making as critical outcomes.

Moreover, among the reviewed studies, the oncology setting was not utilized for FM’s educational interventions. Oncology fellows and radiation oncology residents [26,44] participated in standardized training programs such as Oncotalk and VitalTalk, conducted in specialized communication simulation centers. However, oncologists were not involved in team-based training (physician–nurse) or workplace-integrated learning. Moreover, only three studies [38,40,44] occurred in dedicated palliative care units. These findings suggest training settings remain narrowly focused on acute, institution-bound contexts rather than the diverse environments where palliative communication occurs.

Methodological heterogeneity was evident in sample sizes, training durations, and evaluation frameworks. While quantitative measures predominated, the integration of qualitative data [22,23,24,25,29,31,37,43,45] in select studies enriched interpretations of training acceptability and experiential outcomes. The widespread adoption of simulation-based training underscores its perceived utility in communication education, though variability in assessment rigor suggests the need for standardized evaluation protocols. The limited use of longitudinal follow-up [22,33,35,38,43] highlights opportunities to strengthen causal inferences and assess long-term training impacts in future research.

## 5. Strengths and Limitations

The limitations of this scoping review should be acknowledged. First, our study was restricted to peer-reviewed articles published in English, Italian, and Spanish, potentially excluding relevant studies in other languages. Only peer-reviewed studies were included. Second, literature via other sources, such as clinical trial registers or pre-print databases, was not searched, so educational interventions described in gray literature may have been missed. Lastly, we observed a scarcity of longitudinal studies, which hinders the ability to assess the long-term impact of FMs’ training on HPs’ practices. Future research should address these gaps by incorporating more diverse cultural contexts and conducting follow-up evaluations to better understand the enduring effects of such training.

## 6. Implication

We suggest that FMs’ training should be organized in a multidisciplinary approach, using active learning methodologies, such as simulation-based training and role-playing. Moreover, training should consider not only medical students but also the HPs involved in the assistance routine. Finally, a longitudinal evaluation is desirable.

## 7. Conclusions

We implemented a systematic scoping review to provide a comprehensive overview of FMs’ training programs designed for hospital HPs, and to acquire skills for conducting and/or participating in FMs. The present scoping review defined the extent and pinpointed gaps in training opportunities. This preliminary work is often essential to establish the probably active components of complex educational interventions, the objective of our future study.

## Figures and Tables

**Table 1:** Characteristics of the included studies based on the PCC (Population-Concept-Context) framework.

First Author, Year, State	Objective	Study Design	Trainer(s)	Trainees	Setting	Duration	Teaching Methods	Theoretical Framework	Quantitative Evaluation	Quantitative Outcomes	Qualitative Evaluation	Qualitative Outcomes
**Khawand-Azoulai et al., 2024 USA** [20]	To provide an overview of a curriculum focused on end-of-life discussions and report outcomes from an advanced preparation assignment and student evaluations.	Pre/post interventional study.	Palliative medicine physicians and fellows, geriatricians, nurses (geriatrics and palliative), a social worker and a psychologist (number unspecified).	Medical students (*n* = 80) at the University of Miami Miller School of Medicine (attending final year and participating in a “Transitioning to Residency” course).	University of Miami Miller School of Medicine, Miami, FL, USA (Division of Geriatrics and Palliative Medicine, Department of Medical Education).	Embedded within a 2-week course, the specific intensive training comprises 2 h of pre-session preparation and 1 h of virtual session.	- Virtual, simulation-based session; - In-group lecture (pre-COVID).	Simulation-based learning with interprofessional education (IPE) elements. Program: - Review of online modules and a resource guide; - Completion of a reflection survey; - Small group virtual session (3–4 students) simulation of a family meeting; - Debriefing after the simulation.	- Pre-session reflection survey; - Post-session evaluation survey (five-point Likert scale).	- Agreement on patient’s poor prognosis and shared decision-making; - Anticipated difficulty accepting prognosis, discordance, and challenging emotions; - Post-session evaluations.	N/A	N/A
**Grant et al., 2021 USA** [21]	To describe an interprofessional education event (IPE) for PA (Physician Assistant) students and chaplain residents; To examine whether participating in the IPE event is associated with improvements in attitudes and knowledge. regarding interprofessional teams; To describe participant perceptions about the event.	Pre/post interventional study (with two cohorts).	Faculty team that included PAs and chaplains with experience in simulation design, critical care, advance care planning, and education expertise (number unspecified).	PA students (*n* = 171) and chaplain residents (*n* = 20).	Simulation labs at Wake Forest School of Medicine: Emergency department and Family Conference scenarios.	Two-day event, conducted across two years Day 1: Clinical case (60 min) + debrief (10 min) Day 2: Family conference (25–30 min) + large-group debrief.	In-group simulations Role-play.	In-person Simulation-based IPE Program: Day 1 Simulated cases in an emergency department setting with a high-fidelity mannequin; Day 2 Simulated family conference focusing on delivering poor prognosis and end-of-life decision-making, addressing spiritual dissonance.	Surveys: Five-point Likert scale (pre/post) assessing confidence in prognosis delivery, IPE value, chaplain/PA roles.	- PA students’ confidence in provider–patient communication at the end of life; - Chaplain data on knowledge of the PA role and likelihood of consulting with PAs.	N/A	N/A
**Nagpal et al., 2021** **USA** [22]	To train internal medicine (IM) residents in goals-of-care (GOC) conversations near end of life using Simulation with standardized patients (SPs); Real-life Mini-CEX evaluations with patient/family feedback.	Pre/post interventional study with follow-up (no control group.	Faculty: Six total (hospitalists and/or palliative care clinicians with teaching experience), with three facilitating per session.	Second-year IM residents (*n* = 84).	Simulation Center at University of Massachusetts Medical School: Exam rooms (simulated hospital setting with video recording). Bedside: Real patient encounters during inpatient rotations (Mini-CEX evaluations).	Simulation session: 3 h (resident training) Preparatory meetings: 2 h (for SPs and faculty, held separately).	In-person didactic; Simulation; Real patient encounters.	In-person Didactic: interactive lecture on prognostication/GOC frameworks; Simulation: SP encounters with faculty-paused “time-outs” for feedback; Real-world application (Mini-CEX): observed real patient encounters with faculty feedback (goals-of-care conversations using VitalTalk and the Serious Illness Care Program).	Pre- and post-session self-assessment surveys (follow-up surveys at 1, 3, 6, and 12 months); Mini-CEX (faculty-rated skills); Patient surveys.	- Self-confidence in discussing hospice/prognosis; - Skills (empathy/emotion recognition in Mini-CEX); - Patient feedback.	- Written feedback from residents and patients (comments from program evaluations and post-survey responses); - Debrief sessions post-simulation.	Resident-reported themes: Value of structured frameworks for GOC conversations; Increased comfort with honesty and hospice discussions; Simulation limitations: some found SP interactions “artificial”.
**Donesky et al., 2020** **USA** [23]	To report on the development, exploratory outcomes, and lessons learned from a pilot project, TeamTalk, which taught team-based communication skills using an adapted VitalTalk training methodology.	Pre/post study with qualitative data (no control group).	Interprofessional faculty team trained as VitalTalk facilitators (number unspecified).	Nurses, chaplains, and physicians (61 learners over two years).	- Health sciences university classrooms (University of California, San Francisco); - Simulation center for high-fidelity patient/family encounters.	Two years of course - Year 1: Three sessions (10 h total); - Year 2: Five sessions (10.5–12 h total) over 4–5 months.	High-fidelity simulations (role-play with actors); Skills/capacities handout: adapted from VitalTalk for team communication.	Interactive workshop with VitalTalk methodology; Interprofessional team development and communication skills training.	- JeffSATIC (Jefferson Scale of Attitudes Toward Interprofessional Collaboration); - Self-efficacy survey (19 items, 0–10 scale) on communication confidence.	Interprofessional collaboration attitudes and self-reported confidence.	- Written learner feedback and faculty debriefs; - Thematic analysis by two authors.	Interprofessional dialog; skill development; role clarity across professions.
**Glod et al., 2020** **USA** [24]	To address the problem of insufficient family meeting communication skills training by developing a curriculum for graduate medical trainees that provided learning around facilitation of family meetings during the MICU rotation.	Pre/post interventional study (no control group).	Members of the palliative care service, a social worker, a nurse care coordinator, and an ICU attending or ICU fellow (number unspecified).	34 internal medicine residents.	Medical ICU (MICU) at Penn State College of Medicine’s academic hospital.	Full curriculum cycle: 12 months; Part 1: 1 h; Part 3: 30 min.	In-person didactic; Interactive simulations; Real-world family meeting facilitation; FM resource booklet.	Multimodal Part 1: Introductory interactive session Part 2: Interactive computer-based modules Part 3: MICU introduction session Part 4: family meeting facilitation with self-reflection, peer feedback, and self-assessment.	- CSAS (Communication Skills Attitude Scale): pre/post surveys; - FMBS (Family Meeting Behavioral Skills); Tool: peer feedback during meetings; - Global Self-Efficacy Survey: year-end assessment.	- CSAS scores and self-perceived efficacy; - Frequency of FM (family meeting) facilitation.	Self-efficacy survey (open-ended questions).	- Preparedness (premeeting needs); - Use of silence; - Family-centered mindset; - Feedback barriers (time limitations).
**Manu et al., 2020** **USA** [25]	To improve internal medicine residents’ confidence and skills in addressing eating problems in advanced dementia through an interactive seminar featuring a trigger video and small-group discussion.	Pre/post interventional study (no control group).	Faculty clinician educators: geriatrics/palliative care experts (number unspecified).	IM/medicine-pediatrics/neurology residents (*n* = 82 of 106 participants).	University of Michigan Medical School (IM/medicine-pediatrics residency program); Medical school classroom.	Monthly seminar; video and small-group discussion: 90 min.	Video simulation; In-person case-based learning; In-group discussion; Facilitator guide and participant handouts.	Multimodal Trigger video (14 min): simulated family meeting with actors; Case-based learning: written case study; Small-group discussion; Handouts: evidence summaries.	Pre/post survey using Likert scale (1–4) to assess perceived independence.	- Skill confidence; - Learner satisfaction; - Educational feasibility.	Open-ended text feedback from participants (thematic analysis).	Clinical relevance, group dynamics, seminar format/video quality.
**Cannone et al., 2019 USA** [26]	To improve communication skills in trainees, specifically in oncology, palliative care, and hospice settings, by providing a safe environment to learn and practice these skills.	Pre/post interventional study.	Faculty members: attending physicians in the hematology and oncology, pediatric hematology and oncology, and hospice and palliative care programs. Nine total	Palliative and oncology fellows and radiation oncology residents (*n* = 22)	Educational setting: a classroom for didactic sessions and small meeting rooms for role-play exercises. OSCEs (Simulation Center) Location: Center for Learning and Innovation (CLI), Lake Success, NY.	Overall: 8–9 weeks (2 months). Specifics: Weekly 2 h sessions; Pre/post OSCEs (30 min encounters).	- In-group didactics; - Role-play	Multimodal didactic modules: Eight PowerPoint sessions; Role-play: longitudinal “hot-seat” scenarios with faculty acting as the same patient; OSCEs: pre/post videotaped assessments with standardized patients (SPs).	- Pre/post course self-rated proficiency (four-point scale) surveys; - Objective structured clinical exam (OSCE) with standardized patients (SPs).	- Perceived competence in communication tasks; - SP feedback on OSCEs; - Global communication skills.	N/A	N/A
**Douglas et al., 2018 USA** [27]	To develop a simulation-based training program to teach neurology residents how to accurately diagnose brain death and effectively communicate this diagnosis to the patient’s family with empathy.	Prospective, pre/post interventional study (no control group).	Three neurology attending physicians; 1–2 palliative care attending physicians; Support staff (Simulation nurse and technicians).	18 neurology residents over three years.	Loyola University Medical Center (Chicago).	Three half-days: 4 h pre/post intervention assessments and 5 h didactic intervention.	- In-person lecture; - Role-play.	Technical training (1 h lecture): AAN brain death guidelines, exam maneuvers, and apnea test protocols; Communication training (4 h) - Lecture (2 h): Frameworks like SPIKES, NURSE, and Ask-Tell-Ask; - Role-playing (2 h).	Clinical skills checklist (15 items); Apnea test checklist (9 items); communication skills checklist (37 items). Limitation: Checklists unvalidated (author-developed).	Technical and communication skills.	N/A	N/A
**Awdish et al., 2017 USA** [28]	To determine the feasibility of using a communications bundle to improve patient/family satisfaction and to assess whether the bundle impacted trainee self-perception of communication skills in end-of-life situations.	Prospective cohort feasibility study with control group (pilot).	Physicians, nurses, and fellows trained in VitalTalk and the communications bundle (number unspecified).	MICU Staff (physicians, fellows, residents).	Hospital Medical Intensive Care Unit (MICU).	Overall: 2 weeks.	- Small-group simulation workshops (role-play with actors); - Structured Clinical workflow (post-training); - Mobile-app support.	Four-step bundle: Schedule meeting ≤72 h of admission; Pre-meeting huddle (set agenda/goals); Conduct meeting; Post-meeting huddle (feedback).	- Patient/family satisfaction survey (five-point Likert scale); - Trainee self-perception survey (five-point Likert scale).	- Patient/family satisfaction and trainee self-perception of communication skills in the intervention group compared to the control group.	N/A	N/A
**Hagiwara et al., 2017 USA** [29]	To describe the development and results of a training and assessment program about leading a family meeting.	Single-arm educational intervention with pre/post assessment.	Palliative care faculty preceptors; standardized patient (SP) actors; Clinical skills center staff (number unspecified).	674 fourth-year medical students.	University of Texas Health Science Center at San Antonio (UTHSCSA).	Total time:135 min.	Online didactics (60 min); Small-group role-play (1 h); In-person FM-OSCE with actors (15 min/student).	Family meeting leadership training for medical students: - Online communication module; - Small group FM practice with preceptors; - FM-OSCE Assessment (SPs).	FM-OSCE (Family Meeting Objective Structured Clinical Examination) checklist: 15 domains scored on a 1–5 Likert scale.	- Foundational communication (e.g., empathy, setting), - Tailored discussions over family education, spirituality, and information preferences.	Thematic analysis by two independent investigators. Data Sources: Written feedback from faculty observers (narratives from OSCE assessments); Transcripts of group debriefings with students post-OSCE.	Discussing prognosis clearly and directly, explaining palliative care and hospice, avoiding medical jargon, discussing cultural and religious preferences.
**Schmitz et al., 2016 USA** [30]	To develop and test communication skills intervention for surgical residents using videotapes of end-of-life (EOL) and error disclosure (ED) encounters.	Pre/post interventional study with stratified randomization (control group).	Surgery and orthopedic faculty (number unspecified).	72 PGY1 and PGY3 residents from general surgery and orthopedic programs.	Academic medical center (surgery and orthopedic departments) at the University of Minnesota and Mayo Clinic.	Total intervention time: ~7 h (5 online + 2 in-person).	- Online course; - Face-to-face teaching session; - Role-playing.	Multimodal simulation-based training: 10 video-based online modules; two face-to-face sessions (EOL and ED) with faculty, featuring role-playing and feedback. OSCE assessments: pre- and post-test simulations with SPs.	- OSCE checklists: - EOL/ED rating scales (five-point Likert scales + binary checklists); - Online course evaluation (intervention group only).	Total group: no significant treatment effects (low online engagement and brief face-to-face time); Subgroup effects: low-performing residents showed significant improvement.	N/A	N/A
**Sullivan et al., 2016 USA** [31]	To assess the impact of a communication training program on resident skills in communicating with families in an ICU and on family outcomes.	Prospective, single-site educational intervention study (pre/post interventional).	Critical care physicians (number unspecified).	160 internal medicine residents.	Beth Israel Deaconess Medical Center, Harvard Medical School, Boston, MA: Intensive Care Unit (ICU).	4 h total: two 1 h morning sessions and a 2 h afternoon session.	In-group didactics; Role-play; Standardized communication pamphlet.	Multimodal: Interactive discussions; - Role-playing exercises with simulated family members; - Structured communication guides with suggested phrases; - Focus on foundational and advanced communication skills.	- Resident surveys (pre-course, post-course, 3-month follow-up); - Family member surveys rating resident communication; - Observation of resident–family communication.	- Family ratings of informational and emotional needs; - Resident-perceived skills.	- Family member semi-structured interviews; - Open-ended resident survey questions.	Family member themes: - Importance of personal connection and nonverbal communication; - Need for clearer explanations and checking for understanding; - Resident themes: - Value of structured frameworks and role-playing; - Need for advanced training in handling family conflict and code status discussions.
**Whitaker et al., 2016 USA** [32]	To determine the acceptability and feasibility of a procedure-training module for teaching ICU family conferences to residents.	Pilot feasibility study (no control group; no pre/post evaluation).	ICU faculty and fellows (number unspecified).	27 internal medicine residents (15 interns, 12 PGY-3) during ICU rotations.	Medical ICU at a single academic teaching hospital.	One-month ICU rotation per resident.	- Instructional Vitaltalk video; - Supervised FMs; - Online documentation.	Five components module: - VitalTalk video (seven-step framework for conferences); - Two faculty-observed conferences per resident; - Standardized evaluation form (feedback); - Online procedure log (documentation); - EMR (Electronic Medical Record) template for conference notes.	Survey: 10-item anonymous survey (Likert-scale ratings + open comments).	- Feedback on acceptability and feasibility of the training module.	N/A	N/A
**Arnold et al., 2015 USA** [33]	To develop and evaluate a 3-day communication skills workshop for ICU fellows, aimed at improving skills in delivering bad news, conducting FMs, and discussing GOC.	Pre/post interventional study with self-assessment surveys (no control group).	Faculty facilitators: palliative care, critical care, and communication experts (number unspecified).	38 pulmonary/critical care and critical care medicine fellows (first- and second-year) from a single institution.	Off-site 3-day retreat (away from clinical duties).	Three consecutive days	Skills-based workshop modeled after Oncotalk and focused on active learning (role-play simulation, feedback, reflection).	Didactic talks (20 min each) on evidence-based communication frameworks; Faculty demonstrations of skills; Small-group role-playing (5–7 fellows per faculty facilitator) with standardized families; Sequential cases (three encounters per case).	Pre/post surveys: 11 communication skills rated on five-point Likert scales; 1-month follow-up survey (self-reported skill memory).	Self-rated skills (e.g., giving bad news and conducting family conferences).	N/A	N/A
**Hope et al., 2015 USA** [34]	To develop and evaluate a communication skills program for critical care fellows, integrating simulation, didactics, and feedback to improve family meeting proficiency.	Pre/post interventional study (no control group.)	Critical care and palliative care faculty (five attending physicians, three with palliative care fellowship training); Clinician volunteers (nurses, physicians).	31 critical care fellows (28 participated in simulations) over three years.	Montefiore Medical Center at Albert Einstein College of Medicine: division of Critical Care Medicine.	One month per cohort (repeated annually over three years); Two simulation afternoons (beginning and end of month) lasting ~3 h.	- In-person, group-based sessions; - Role-play simulations.	Multimodal learning: Simulation + didactics + real-time feedback; Needs assessment and pre-course trainee survey; Family meeting simulations; Lectures/case discussion; Trainee’s post-course evaluations.	Faculty-rated checklists (nine specific behaviors); Five-point Likert scales (quality of communication in four domains); Pre/post self-report surveys.	Agenda-setting, summarizing care, follow-up plans.	N/A	N/A
**Markin A 2015 USA** [35]	To improve residents’ EOL communication skills via a brief simulation-based workshop (VitalTalk method).	Pre/post interventional (no control group).	Two faculty facilitators (physicians) trained in VitalTalk.	34 s-year internal medicine residents (PGY-2).	Academic medical center ICU at the Henry Ford Hospital (Detroit, MI).	3-day VitalTalk workshops for faculty members; Three 90 min sessions for residents; 9-month follow-up.	- Pre-session written module; - In-person didactic lecture; - Small-group role-play with actors + “rewind” feedback.	VitalTalk curriculum Written module sent one week prior to the session; Short didactic overview of the core skills; Faculty demonstration; Resident practice with a simulated ICU family member.	Pre- and post-survey with five-point Likert scales (self-assessed preparedness).	- Giving bad news to a family; - Conducting a family conference; - Expressing empathy); - Skills sustained at 9 months (except empathy).	N/A	N/A
**McCallister et al., 2015 USA** [36]	To evaluate the effectiveness of a communication skills curriculum for pulmonary and critical care medicine (PCCM) fellows, using formative feedback (Family Meeting Behavioral Skills Checklist) to improve family meeting skills in the ICU.	Prospective pre/post quasi-experimental interventional study (control group).	Palliative medicine faculty supervisors with expertise in communication skills and critical care + psychologists (number unspecified).	11 first-year PCCM fellows (intervention group); 5 s-year fellows (historical controls).	Setting integrated into the first year of PCCM fellowship: academic MICU. Ohio State University Wexner Medical Center, Columbus, OH.	12-month curriculum; 3 h workshop + FMs.	- In-person didactic; - Role-play; - Bedside training.	Multimodal Behavioral skills-based curriculum: Pre-curriculum assessment (baseline surveys + simulated family meeting); Workshop (Didactic lecture and role-play with feedback); Clinical rotations: ≥2 supervised real ICU family meetings per rotation (four rotations/year); Post-curriculum assessment (Simulated family meeting and self-confidence surveys).	- Educational experience and attitudes questionnaire; - Self-confidence in communication skills survey; - Family Meeting Behavioral Skills Checklist (FMBSC).	Behavioral skills and confidence.	N/A	N/A
**Milic et al., 2015 USA** [37]	To improve critical care nurses’ skills and confidence to engage in discussions with patients’ families and physicians about prognosis and goals of care by using a focused educational intervention.	Pre/post interventional (no control group).	Interdisciplinary working group: ICU bedside nurses, a critical care nurse researcher and educator, a palliative care physician, a critical care and palliative care physician, and a palliative care chaplain and psychologist.	82 critical care nurses, 15 per workshop.	Academic medical center: University of California San Francisco Medical Center.	8 h workshop; 3× role-plays (60–70 min each).	- Didactic lecture; - Role-play; - Reflection session.	Communication skills-focused: Brief didactic session (roles of nurses: “The Four Cs”); Three role-play discussions; Reflection session (combatting burnout); Surveys before, immediately after, and 3 months after the workshop.	Four-point Likert-type scale for 14- to 22-item surveys.	- Skills (nine items), sustained at 3 months; - Confidence (five items).	Focus group (*n* = 11) + open-ended survey responses. Thematic analysis of impact.	Themes: - Role clarification; - Toolkit; - Empowerment; - Increased empathy; - Culture change.
**Gelfman et al., 2014 USA** [38]	To evaluate the effectiveness of the Geritalk communication skills course by comparing pre- and post-course real-time assessment of participants leading family meetings and to evaluate participants’ sustained skills practice.	Pre/post interventional (no control group).	Six course faculty: physicians on the Geriatric Medicine and Palliative care hospital services.	Nine first-year fellows (five palliative medicine, four geriatrics).	Academic medical centers: Mount Sinai Medical Center and the James J. Peters Bronx VA Medical Center.	Overall: 2-day workshop + 2-month follow-up. Specifics: Didactics (4 h) + small-group simulations (8 h). Clinical assessments: Pre/post family meetings (~42 min each).	- Didactics (in-person); - Small-group (simulations and FMs).	Didactic-experiential: Baseline survey; Pre-course family meeting assessment; Geritalk course; Post-course family meeting assessment; Post-course self-assessment.	- FaMCAT (Family Meeting Communication Assessment Tool) on a five-point Likert scale (81-item checklist) - Surveys: Self-rated competence/confidence (Likert scales); - Deliberate practice tracking at 2 months.	- Skill acquisition (fundamental and advanced); - Deliberate practice feedback after the post-course family meeting assessment.	N/A	N/A
**Hauer et al., 2013 USA** [39]	To describe a pilot and feasibility evaluation of two Entrustable Professional Activities (EPAs) for competency-based assessment in internal medicine (IM) residency.	Pilot feasibility study (pre/post intervention design with surveys). No control group.	Leadership group: Residency program director, associate chair for education, chief residents, and faculty with medical education expertise. Faculty developers: Palliative care faculty (for family meeting EPA); hospital medicine faculty (for Discharge EPA). (number unspecified).	26 PGY-1 internal medicine residents (involved in the family meeting EPA assessment and related surveys).	General: University of California, San Francisco (UCSF) IM residency program. Particular: - Discharge EPA: Inpatient wards at university hospital. - Family meeting EPA: Palliative care rotation at Veterans Affairs hospital.	Discharge EPA Overall: Integrated into four-week rotation. Specifics: Didactics (1 h discharge summary lecture, 2 × 1 h noon conferences). Family meeting EPA Overall: 1-week rotation. Specifics: 1 h noon conference, online modules, critical reflection exercise.	- In-person lecture; - Self-directed learning (observation, reflection, written feedback).	Multimodal (didactic + workplace-based assessment): - Discharge EPA: Didactics, peer review of discharge summaries, rubric-guided feedback. - Family Meeting EPA: Observation, reflection, written feedback.	Surveys (Likert-scale questions); Completion rates of EPA assessments.	- Survey response rates; - EPA assessment completion rates; - Participant satisfaction (Likert scales); - Perceived skill improvement (from surveys, e.g., patient management, communication); - Usefulness of feedback (from surveys); - Recommendations for continuing the EPA (from surveys).	N/A	N/A
**Kelley et al., 2013 USA** [40]	To develop and evaluate an intensive communication skills training course for geriatrics and palliative medicine fellows (Geritalk).	Single-arm pre/post interventional (no control group). Pilot study with mixed-methods evaluation (surveys + open-ended feedback)	Six course faculty trained in small-group facilitation techniques: attending physicians on the Geriatric Medicine and Palliative Care hospital services.	Geriatrics and palliative medicine fellows (*n* = 16).	N/A	Overall: 2 days Specifics Large-group didactics: 25 min lectures (four sessions). Small-group practice: 2.5 h sessions (four sessions).	In-group didactics; In-person (one-on-one mentorship); Role-play.	Simulation-based retreat drawn upon the Oncotalk method. Overview presentations; Skills practice; Future skills practice commitment.	Surveys: Pre/post self-assessed preparedness (five-point Likert scale); Post-retreat satisfaction (five-point Likert scale): 2-month follow-up on skills practice frequency.	Overall satisfaction; Self-assessed preparedness for communication challenges; Sustained skills practice.	N/A	N/A
**Shaw et al., 2013** **USA** [41]	To train multidisciplinary teams of ICU caregivers in communicating with the families of critically ill patients, to improve staff confidence as well as family satisfaction.	Pre/post interventional study (no control group).	N/A	98 ICU physicians and hospital staff (intensivists, medical residents, ICU chaplains, nurses, social workers, respiratory therapists, pharmacists, and case managers).	Community hospital (Scripps Mercy Hospital, San Diego) medical and surgical ICUs.	Overall: 14 sessions conducted between May–September 2011. Specifics: 90 min sessions (didactic + simulation + debrief).	- In-person lecture; - Role-play sessions; - In-group debriefs.	Multidisciplinary team training in a four-step bundle: Pretraining reading (SPIKES protocol + ICU family testimonials); Didactic SPIKES review; Role-play simulation (standardized family conference); Debrief with self-assessment.	- Provider confidence survey; - Family satisfaction survey using Family Satisfaction in the ICU 24 (FS-ICU 24); - 10-point ordinal scale to measure staff confidence before and after the training	Staff confidence in communication increase (21 skills measured); Family satisfaction.	N/A	N/A
**Tchorz et al., 2013 USA** [42]	To ensure exposure to the communication skills needed to effectively manage complex palliative and end-of-life care scenarios commonly encountered during each surgical clerkship rotation.	Pre/post interventional (single-group, no control).	Faculty members (debriefing facilitators): One ethicist; one geriatrician. Student evaluators: Seven full-time WSU-BSOM surgeons.	97 Wright State University Boonshoft School of Medicine (WSU-BSOM) third-year medical students, during their surgical clerkship.	Wright State University Boonshoft School of Medicine (WSU-BSOM): clinical settings included Trauma, Intensive Care Unit, Transplant, and Surgical Oncology clinical services (from Skills and Assessment Training Center).	Overall: 8-week surgical clerkship; Specific: OSCEs conducted during Week 4 of the 8-week clerkship; 1 h for all six OSCE stations; 10 min per OSCE station (student–patient interaction), 2 min between stations. 1 h group debriefing after all OSCEs.	- Standardized patient encounters. - In group debriefing sessions. - Online modules + pocket guides.	Multimodal didactic (online modules) + simulation (OSCEs): 1) Presentation of six palliative/end-of-life case scenarios with educational material and best practice guideline (websites); 2) Three laminated reference pocket cards for communication phrases; 3) Teaching video; 4) Six Objective Structured Clinical Examination (OSCE); 5) Debriefing session by an ethicist and a board-certified geriatrician.	- Binary checklist scoring; - Passing threshold (71% of tasks).	Mean percent scores for each of the six OSCE scenarios; Total score for the six scenarios.	N/A	N/A
**Downar J et al., 2012 Canada** [43]	To determine the effectiveness of standardized family members (SFMs) for improving communication skills and ethical/legal knowledge of senior ICU trainees.	Multimodal evaluation of mixed-methods educational intervention (Pre/post quantitative tests + planned qualitative debriefing/feedback).	Staff intensivists, communication skills educators, and standardized family members (SFMs) from the standardized patient program at the University of Toronto (number unspecified).	51 Postgraduate subspecialty Critical Care Medicine trainees.	Postgraduate Critical Care Medicine academic program at the University of Toronto.	Half-day workshop; 60 min didactic session; Four 45 min simulated family meetings; 15 min for feedback and completion of the evaluation forms; 1-yr follow-up scenario; 5-year period workshop.	- In-person (Short didactic session); - Role-play (four simulated family meetings using trained professionals as standardized family members); - In group feedbacks	Multimodal didactic + simulation - Didactic session and printed material covering key communication principles and schemata from the literature; - Four 45 min simulated family meetings with standardized family members (SFMs).	- Pre- and post-workshop tests: 11-question test on ethical/legal principles; - “needs assessment” (nine questions on comfort, five-point Likert scales); - 16-aspect trainee performance scale (self-evaluation); - survey at 1–4 years post-workshop.	- Ethical and legal knowledge; - Comfort with communication challenges; - Communication skills.	- Structured debriefing: Post-scenario feedback sessions; - 1-year follow-up survey: Open-ended reflections on real-world application.	- Workshop effectiveness; - Emotional preparedness; - Long-term impact.
**Gueguen ja et al., 2009 USA** [44]	To develop and evaluate a communication skills training module for healthcare professionals on conducting family meetings in palliative care, assessing self-efficacy and satisfaction post-training.	Pre/post interventional study (retrospective).	Communication Skills Training and Research Laboratory (Comskil) at Memorial Sloan-Kettering Cancer Center (MSKCC). The final author (psychiatrist D.W.K.) facilitated a role-play.	40 multi-specialty healthcare professionals (medical/surgical/radiation oncology, pediatrics, palliative care); Nurses/nurse practitioners, physician assistants.	Communication Skills Training and Research Laboratory (Comskil) at MSKCC and other New York City Metropolitan-area hospitals.	Two-day course for 77% of participants (those new to the Comskil curriculum).	In-person didactic presentation and “fishbowl” role-play with simulated family members.	Multimodal didactic + simulation Booklet; Didactic teaching; Exemplary video; Role-play.	Retrospective pre/post surveys (Likert three- and five-points scales).	- Self-efficacy in conducting family meetings, - satisfaction with training.	N/A	N/A
**Chipman JG et al., 2007 USA** [45]	To describe the development and results of an Objective Structured Clinical Exam (OSCE) for leading family conferences in the surgical intensive care unit (SICU).	Pilot demonstration and reliability assessment.	Surgical Critical Care Faculty; Interdisciplinary Raters: Four ICU nurses, one neurologist, two additional surgeons, one educator.	PGY-2 and PGY-4 categorical general surgery residents (*n* = 8)	University of Minnesota Medical School (academic teaching hospital): Inter-professional Educational Resource Center (IERC) with standardized exam rooms and video recording.	OSCE Session Duration per resident: Two 20 min stations + 45 min pre-lecture. The entire testing period: 4 h.	- In-person lecture; - Role-play.	Simulation-based OSCE Pre-OSCE prep residents: didactic on communication skills. Actors: training to standardize emotional responses (anger, grief). Core components two simulated stations: end-of-life discussion and complication disclosure.	Two rating scales (EOL + Disclosure): four-point Likert items and dichotomous checklists.	- Reliability: internal consistency of the data/inter-rater reliability; - Performance: PGY-2 vs. PGY-four differences.	- Post-OSCE debriefs; - Raters written comments; - Evaluation surveys.	Resident feedback themes: Realism vs. simplification; Scenario critique actor/rater observations and work group reflections.

N/A not available.

## Data Availability

The authors confirm that the data supporting the findings of this study are available within the article.
